# Refining global HIV estimates for decision‐making: advances in analytic and modelling methods used by the Joint United Nations Programme on HIV/AIDS

**DOI:** 10.1002/jia2.25790

**Published:** 2021-09-21

**Authors:** Mathieu Maheu‐Giroux, Andrea L. Ciaranello, Joshua A. Salomon, Annette H. Sohn

**Affiliations:** ^1^ Department of Epidemiology and Biostatistics School of Population and Global Health McGill University Montréal Québec Canada; ^2^ Division of Infectious Diseases and Medical Practice Evaluation Center Massachusetts General Hospital Harvard Medical School Boston Massachusetts USA; ^3^ Center for Health Policy Center for Primary Care & Outcomes Research School of Medicine Freeman Spogli Institute for International Studies Stanford University Stanford California USA; ^4^ TREAT Asia/amfAR – The Foundation for AIDS Research Bangkok Thailand

**Keywords:** AIDS, epidemiology, estimates, global, HIV, indicators, modelling, projections, surveillance, UNAIDS

## 1 INTRODUCTION

The annual global HIV estimates, produced through the contributions of national surveillance programs and published by the Joint United Nations Programme on HIV/AIDS (UNAIDS), are the world's primary source of aggregated HIV surveillance and program data (aidsinfo.unaids.org). They provide critical metrics that guide implementation of regional and global public health programs, allocation of international funding, community advocacy and research. To generate these estimates, UNAIDS and its partners support the development of tools, models and software, and provide guidance to countries on how to use them. The latest estimates from 2020 were released in July 2021, after substantial efforts to mitigate the negative impacts of the COVID‐19 pandemic on this annual estimation process, including establishing remote data support platforms for local teams [1].

As affected populations and the coverage of prevention and treatment programs shift over time, additional modelling and analytical techniques have been developed in collaboration with the UNAIDS Reference Group on Estimates, Modelling and Projections (epidem.org) to more accurately capture trends in HIV testing, prevention and treatment outcomes in specific geographic and population contexts. This supplement, the latest installment in a biennial series, highlights major advancements in these methods, explains how they have been developed and shares recommendations around future research.

## 2 GLOBAL HIV CONTROL: A MIXED PICTURE BY LOCATION AND POPULATION

Mahy et al. [2] remind us that countries devote extensive resources to measuring new HIV infections, AIDS‐related deaths and progress towards the United Nations targets as accurately as possible, and how critical these efforts are to understanding an increasingly heterogeneous pandemic. Updated analyses show that while considerable progress has been made, there are growing gaps between regions and countries, as well as across sub‐populations. For example, new global HIV infections fell by 31% and AIDS‐related deaths by 47% between 2010 and 2020, but two regions saw HIV infections rising over this period (eastern Europe and central Asia, Latin America, Middle East and North Africa), and one region had an increase in deaths (eastern Europe and central Asia). By the end of 2020, UNAIDS 90‐90‐90 global cascade indicators reached their highest estimated levels at 84‐87‐90, an achievement that was supported by numerous local stakeholders and funders, including the US President's Emergency Plan for AIDS Relief (PEPFAR) and the Global Fund to Fight AIDS, Tuberculosis, and Malaria. At the same time, a small number of individual countries achieved the testing and treatment targets. Moreover, the cascade values were worse for men than women (82‐83‐91 vs. 88‐90‐91), and were only 59‐91‐75 for children <15 years of age. Few countries have suitable data to separately calculate them for vulnerable key populations who experience stigma and discrimination that compromises access to services. Importantly, the uncertainty ranges for these estimates are large, indicating that the degree to which key targets have been missed may be greater than suggested in the results most frequently cited. Additional papers in this supplement provide insights into the UNAIDS Reference Group’s plans for how they will improve methods for estimating future progress in sub‐populations.

## 3 CRITICAL MODELLING UPDATES AND MODIFICATIONS TO ESTIMATION TOOLS

Models and methods underpinning the estimation of core HIV epidemic projections are continually improved in response to emerging data streams, evolving transmission dynamics and strategic needs of program managers and decision makers. The Spectrum model forms the backbone of the global HIV modelling framework and allows countries to use different estimation tools, reflecting contrasting data availability and epidemic settings [3].

One of the key tools within Spectrum is the AIDS Impact Module (AIM). Stover et al. [4] describe some of the major updates to AIM. First, new data from the International Epidemiology Databases to Evaluate AIDS (IeDEA) global consortium were used to improve paediatric HIV parameters, a new meta‐analysis was conducted to improve estimates of antiretroviral therapy (ART) discontinuation rates among breastfeeding women and analyses of breastfeeding patterns among women living with HIV were updated. Second, Avenir Health in collaboration with the UNAIDS Reference Group improved how the ART care continuum was modelled by explicitly considering people living with HIV who were previously treated but subsequently disengaged from care. They also revised assumptions around CD4 counts among people who discontinue ART to account for previous CD4 count improvements while on treatment. Third, they considered potential COVID‐19 disruptions to HIV treatment services by allowing countries to report monthly data on the numbers of people on ART, instead of only annual data. Taken together, Stover et al. [4] found that these changes had minor impacts on paediatric indicators, but that their effect on AIDS‐related deaths could vary more widely in specific contexts.

When robust case reports of new HIV diagnosis and AIDS deaths from vital registration systems are the primary data source, such as in settings of low HIV prevalence, the Spectrum *Case Surveillance and Vital Registration* (CSAVR) within the AIM model may be used to estimate HIV indicators. Mahiane et al. [5] present three main improvements to CSAVR. First, they modified how HIV diagnosis rates are estimated. Previous CSAVR versions assumed that diagnosis rates were functions of HIV‐related mortality. Their new approach is more flexible and allows for estimates of knowledge of HIV status to vary by sex and age. Second, for countries with age‐ and sex‐disaggregated data on HIV diagnoses and HIV‐related deaths, HIV incidence rate ratios stratified by sex and age can now be estimated within the model rather than using Spectrum's default ratios. Third, although not included in the current model, CSAVR has been updated to potentially produce estimates of HIV incidence and new HIV diagnoses among four key populations: men who have sex with men, men who inject drugs, female sex workers, and women who inject drugs. Mahiane et al. [5] tested the updated CSAVR model in 71 mostly high‐income countries, and found that knowledge of HIV status was close to 90%. Among countries with data for key populations, knowledge of HIV status exceeded 75% in over 75% of countries.

Understanding HIV epidemic heterogeneity and unmet needs at sub‐national scales is important for program planning but has been challenged by sparse local data. Eaton et al. [6] developed new modelling tools to estimate district‐level (sub‐national) HIV indicators in sub‐Saharan Africa. Specifically, they used a Bayesian small‐area estimation model – named Naomi – to jointly estimate sub‐national HIV prevalence, incidence, HIV knowledge of status and ART coverage by sex and age. The model leverages information from population denominators, HIV seroprevalence surveys, ART service delivery data and HIV testing of pregnant women to produce estimates for the current period at sub‐national scales, and allows for short‐term projections. Although the analyses in their paper highlight considerable within‐country variations, Naomi has been successfully used by 38 countries to date, and the authors suggest opportunities for further development.

## 4 IMPROVING STANDARDIZATION AND VALIDITY OF KEY ANALYTIC INPUTS

An important feature in UNAIDS modelling efforts that support regular updates to epidemiological and programmatic estimates in global HIV has been the development of approaches to improve standardization and comparability of data inputs into the suite of models used for estimation. Three papers in this issue address aspects of this endeavour. The paper by Johnson et al. [7] presents a new adjustment to improve standardization in estimates of the probability of viral suppression among patients on ART – the third 90 in the UNAIDS 90‐90‐90 targets [8]. A challenge in estimating progress towards the third 90 has been the lack of consistent threshold definitions for reporting viral suppression (e.g. <400 copies/ml, <1000 copies/ml). By examining alternative statistical distributions for describing viral load, and fitting distributions derived from each approach to empirical data from two large collaborative networks studying outcomes in ART programmes, Johnson and colleagues [7] identified the best‐fitting model for viral load and proposed new standards for adjusting programmatic data in order to assess viral suppression in a more comparable way across countries.

The Spectrum model relies on estimates of disease progression and mortality risks in the absence of ART (“natural history”) to relate HIV incidence, prevalence and mortality. Previously, these estimates have been based on data from observational cohort studies conducted before the widespread availability of ART. CD4 data from untreated adults with HIV in 10 Population‐based HIV Impact Assessment (PHIA) surveys over the period 2015–2018 provide a new opportunity to anchor disease progression estimates on this additional source of natural history information. Glaubius et al. [9] describe the results from a new evidence synthesis incorporating the PHIA data to update the natural history inputs to Spectrum. The new parameters based on this analysis indicate lower CD4 counts at treatment initiation than previously assumed, with a corresponding increase in the estimated rates of progression through infection stages, and a decrease in most of the stage‐specific mortality rates.

Accurately classifying HIV‐related causes of death is especially challenging in low‐ and middle‐income countries with incomplete death reporting. The CSAVR model, described by Mahiane et al. [5], fits epidemic curves to case surveillance data and cause‐of‐death data in settings where vital registration is nearly complete. Even where deaths are reliably captured, however, variation in cause‐of‐death coding practices can compromise the validity of HIV mortality data without adjustment of these data. Kyu et al. [10] describe the approach used in the Global Burden of Disease (GBD) study to adjust for misclassified cause‐of‐death data. Analysing data from 132 countries over the period 1990–2018, they found that redistribution of deaths from “garbage codes” (codes that incorrectly attribute cause of death to immediate or intermediate causes, such as respiratory failure) to HIV/AIDS increased the number of deaths directly attributed to HIV over this period from 1.8 to 4.2 million, and that reclassifying deaths assigned to other underlying causes, such as tuberculosis and encephalitis, added another 1.9 million deaths to the total. A subset of the 48 countries that use CSAVR also have chosen to use the adjusted GBD mortality estimates as an additional data source for fitting their model.

## 5 HIV IN PREGNANCY AND CHILDHOOD, UPTAKE OF VOLUNTARY MEDICAL MALE CIRCUMCISION AND IMPLICATIONS FOR ESTIMATES OF VERTICAL AND SEXUAL TRANSMISSION

As noted by Stover et al. [3], Spectrum‐based projections of global paediatric HIV estimates depend importantly on several key factors. The number of women with HIV who become pregnant each year determines the projected number of children at risk for and acquiring HIV, which in turn impacts the estimates of children in need of ART and number of deaths among children and adolescents with HIV. An influential factor in this stream of modelled events is at the most upstream level: the number of women who acquire HIV during pregnancy. Incident infection during pregnancy poses a substantial risk of intrauterine or intrapartum transmission to infants. Previous data suggested that women are at increased risk of acquiring HIV per coital act during pregnancy compared to when they are not pregnant, due to a combination of hormonal and other physiologic factors, but it was not known whether reductions in sexual activity during pregnancy might offset this risk. Marston et al. [11] report a novel analysis of data from Demographic and Health Surveys from 32 sub‐Saharan African countries, and find that sexual activity was reduced in the last few months of pregnancy in most settings. At the population level, the total risk of acquiring HIV during periods of high risk for vertical transmission was, therefore, less than would be estimated from per‐coital‐act risks alone. The incorporation of these revised risks into Spectrum projections was identified as an area for future model refinement.

The projected survival of children on ART also substantially influences Spectrum results. Yearly global estimates of those surviving and eligible for ART serve as the denominator for estimates of ART coverage, with program data as the numerator. In addition, projected deaths among children and adolescents on ART comprise an important component of total estimated paediatric and adolescent mortality. In 2016, Spectrum substantially revised its estimates of on‐ART mortality for children and adolescents through a collaboration with the IeDEA consortium [12]. In this issue, Kassanjee et al. [13] updated these IeDEA mortality estimates using more recent data, as well as findings from a novel tracing study that assessed outcomes among children who were lost to follow‐up from IeDEA programs. The adjustment they used to account for very high mortality among children no longer in care resulted in important refinements to the estimates for the entire population in their analysis, diminishing the estimated improvement in mortality between 2005 and 2017 in this test environment (from an unadjusted decline of 60% to a more modest adjusted decline of 42%).

Beyond models to monitor HIV epidemic trends, tools to access, curate and use quality HIV program data are essential for program planning and management. Increasing coverage of voluntary medical male circumcision (VMMC) in countries prioritized by UNAIDS and the World Health Organization (WHO) for HIV control efforts has been recommended to prevent HIV transmission. Stegman et al. [14] describe the development and validation of the Decision Makers Program Planning Toolkit (DMPPT) 2, an online tool to support countries to collect VMMC data in a standardized format to align with Spectrum and Naomi's subnational models. The integration of the DMPPT2 Online into Spectrum improves capacity to set age‐ and geographic‐specific VMMC targets, and track program implementation.

## 6 CONCLUSIONS

Although the 2020 global HIV testing and treatment targets reached estimated levels of 84‐87‐90 [2], progress remains heterogeneous across regions and populations. Monitoring national and global efforts to end HIV epidemics relies on robust surveillance data and internationally comparable indicators. Central to the UNAIDS‐supported country‐led estimation process is the annual feedback gathered from national HIV programs, which has resulted in a broader range of estimates and greater disaggregation by age, sex and sub‐populations.

The papers in this supplement provide a look inside the model development process. The improvements to the Spectrum model include adjustments to multiple assumptions around HIV natural history, care disengagement and mortality in children and adults. Country data reporting requirements were revised to accommodate COVID‐19 pandemic disruptions on HIV care delivery. Important updates to CSAVR and the new Naomi model will both allow for estimates at finer levels of stratification. In addition, the UNAIDS Reference Group is developing ways to harmonize data sources and collection tools in collaboration with implementers, researchers and other stakeholders to help standardize estimates.

These methods must continue to adapt to evolving epidemiological and public health contexts, with regular reassessment of parameters and assumptions, and development of new tools to improve the precision and accuracy of HIV estimates. While this may ultimately result in substantial revisions to previous estimates, the updated models will more accurately characterize the unmet treatment and prevention needs of individuals for countries to end AIDS.

## FUNDING

The supplement “Advancing Methods for Global HIV Estimates” was supported by UNAIDS. MM‐G's research program is funded by a Tier 2 Canada Research Chair in Population Health Modeling.

## COMPETING INTERESTS

AHS receives grants to her institution from ViiV Healthcare.

## AUTHORS' CONTRIBUTIONS

MMG, ALC, JAS and AHS contributed to writing, revising and finalizing the editorial. All authors have read and approved the final manuscript.

## DISCLAIMER

The content of this publication is solely the responsibility of the authors and does not necessarily represent the official views of any of the institutions mentioned above.
